# Coronin 1B Regulates S1P-Induced Human Lung Endothelial Cell Chemotaxis: Role of PLD2, Protein Kinase C and Rac1 Signal Transduction

**DOI:** 10.1371/journal.pone.0063007

**Published:** 2013-05-08

**Authors:** Peter V. Usatyuk, Michael Burns, Vijay Mohan, Srikanth Pendyala, Donghong He, David L. Ebenezer, Anantha Harijith, Panfeng Fu, Long Shuang Huang, James E. Bear, Joe G. N. Garcia, Viswanathan Natarajan

**Affiliations:** 1 Institute for Personalized Respiratory Medicine, University of Illinois, Chicago, Illinois, United States of America; 2 Department of Pharmacology, University of Illinois, Chicago, Illinois, United States of America; 3 Department of Pediatrics, University of Illinois, Chicago, Illinois, United States of America; 4 Department of Cell and Developmental Biology, University of North Carolina at Chapel Hill, Chapel Hill, North Carolina, United States of America; 5 Department of Medicine, University of Illinois, Chicago, Illinois, United States of America; University of Birmingham, United Kingdom

## Abstract

Coronins are a highly conserved family of actin binding proteins that regulate actin-dependent processes such as cell motility and endocytosis. We found that treatment of human pulmonary artery endothelial cells (HPAECs) with the bioactive lipid, sphingosine-1-phosphate (S1P) rapidly stimulates coronin 1B translocation to lamellipodia at the cell leading edge, which is required for S1P-induced chemotaxis. Further, S1P-induced chemotaxis of HPAECs was attenuated by pretreatment with small interfering RNA (siRNA) targeting coronin 1B (∼36%), PLD2 (∼45%) or Rac1 (∼50%) compared to scrambled siRNA controls. Down regulation PLD2 expression by siRNA also attenuated S1P-induced coronin 1B translocation to the leading edge of the cell periphery while PLD1 silencing had no effect. Also, S1P-induced coronin 1B redistribution to cell periphery and chemotaxis was attenuated by inhibition of Rac1 and over-expression of dominant negative PKC δ, ε and ζ isoforms in HPAECs. These results demonstrate that S1P activation of PLD2, PKC and Rac1 is part of the signaling cascade that regulates coronin 1B translocation to the cell periphery and the ensuing cell chemotaxis.

## Introduction

Sphingosine-1-phospahte (S1P) is a bioactive sphingophospholipid that has been shown to enhance endothelial chemotaxis during wound healing [1]. Coronin is one of the actin-regulatory proteins present at the leading edge of migrating cells [2] and has been shown to enhance cofilin-mediated actin de-polymerization [3], [4] and inhibit Arp2/3-mediated actin nucleation [5]. The idea that coronin is a critical protein for efficient cell migration is supported by the literature which reports on the presence of coronin at the leading edge of migrating cells [2], [6], [7], its co-localization with other actin-regulating proteins at sites of rapid actin turnover [8], [9] and the impaired migration of coronin mutant cells [10], [11]. However, the detailed mechanisms of coronin-mediated cell motility are still unclear.

The leading edge, or lamellipodia, of migrating cells exhibits a unique type of actin dynamics characterized by the fast “treadmilling” of actin filaments [12] where F-actin filaments are depolymerized at their pointed ends to liberate G-actin monomers that are recycled to extend F-actin filaments at their barbed end. Rapid actin disassembly is an important aspect of lamellipodia actin dynamics as it replenishes the G-actin monomers necessary for extending F-actin filaments. Compromise of actin depolymerization has been shown in cell models to reduce migration rates. Cofilin is the major actin-regulating protein involved in actin depolymerization by facilitating the removal of ADP-bound G-monomers from the pointed ends of F-actin filaments [13], [14]. However, in the presence of G-actin monomers, cofilin is unable to depolymerize actin without coronin [3]. Although coronin has been identified as a critical cofactor for cofilin, signaling pathways regulating cofilin dephosphorylation by SSH1 and coronin relocalization to leading edges of cells are currently not well defined.

Recently, the role of phospholipase D (PLD) in cell migration has been demonstrated [15], [16], [17]. PLD isoforms 1 & 2 hydrolyze phosphatidylcholine to phosphatidic acid (PA), which is a second messenger and involved in membrane trafficking [18], actin cytoskeleton remodeling [19], [20] and cell survival [21]. Over-expression of catalytically inactive PLD2 in normal endothelial [15] and cancer cells [22] inhibited cell migration, suggesting a role for PLD in regulation of cell motility. The signaling pathways downstream of PLD leading to cell migration have not been clearly defined; however, PA can directly activate PKC ζ [23], and PKC isoforms have been shown to be involved in cell migration in various cell types [15], [24], [25].

We and others have demonstrated that S1P activates PLD in endothelial and other cell types [26]; however, the potential role of PLD in S1P-induced chemotaxis in endothelial cells is not well defined. In the present paper, we investigated the role of coronin 1B and PLD signaling in S1P-induced endothelial cell chemotaxis. Treatment of human pulmonary artery endothelial cells (HPAECs) with S1P rapidly induced coronin 1B localization to lamellipodia and enhanced chemotaxis. Silencing coronin 1B with small interfering RNA (siRNA) attenuated S1P-induced HPAEC chemotaxis. Further, PLD2, PKC δ, ε and ζ and Rac1 signal transduction regulated S1P-mediated coronin 1B localization to lamellipodia and chemotaxis.

## Results

### Expression and Localization of Coronin 1B in Human Endothelial Cells

**Figure 1:**
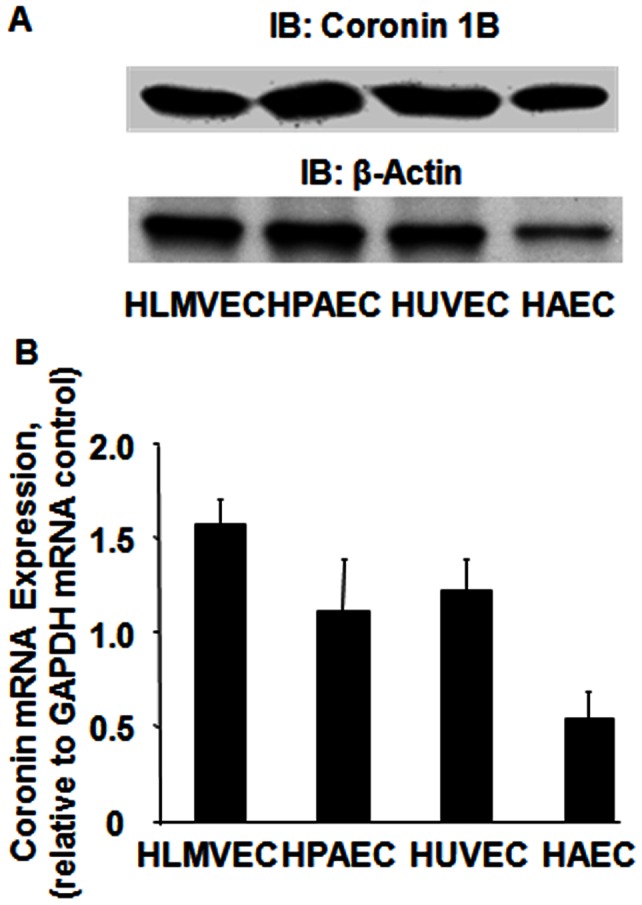
Expression of coronin 1B in human endothelial cells. Human lung microvascular, pulmonary artery, umbilical vein and aortic ECs grown to ∼90% confluence in 60 mm dishes were lysed in cell lysis buffer. Cell lysates (20–40 µg of protein) were subjected to 10% SDS-PAGE, transferred to PVDF membrane and probed with anti-coronin 1B and β-actin antibodies as described under Materials and Methods. (**A**), Shown is a representative Western blot depicting coronin 1B protein expression in different human ECs. (**B**), In parallel experiments total RNA was isolated from various human ECs and were analyzed for mRNA expression of Coronin 1B by quantitative real time RT-PCR. The values are mean ± S.E.M for three independent experiments each g002performed in triplicate and normalized to GAPDH mRNA expression.

**Figure 2:**
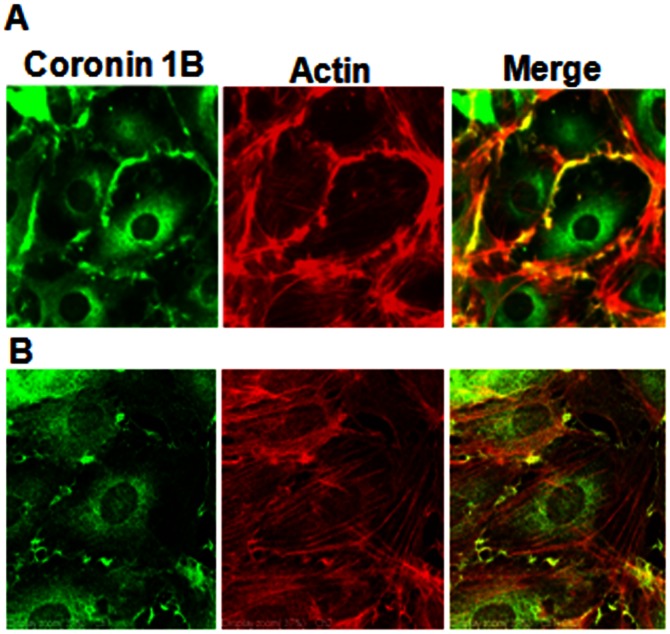
Coronin 1B localization in human lung endothelial cells. HPAECs grown to ∼90% confluence on slide chambers were fixed, permeabilized and localization of Coronin 1B, actin and co-localization of Coronin 1B with actin was visualized by immunocytochemistry as described in Materials and Methods. Shown are representative immunofluorescence images from several independent experiments as measured by regular (**A**) immunofluorescence and (**B**) confocal microscopy.

**Figure 3:**
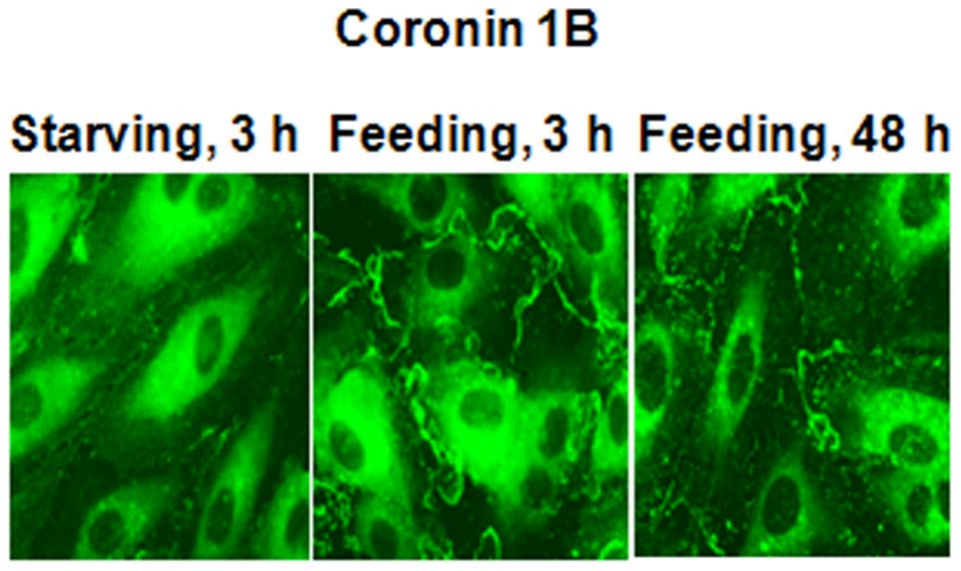
Effect of starvation and serum supplementation on Coronin 1B localization in human lung endothelial cells. HPAECs grown on slide chambers (∼90% confluence) were incubated in EBM-2 medium containing either 0.1% serum for 3 h or in EBM-2 medium containing 5% serum for 3 h and 48 h. Cells were fixed, permeabilized and Coronin 1B localization was visualized by immunocytochemistry as described in Materials and Methods. Shown are representative immunofluorescence images from several independent experiments.

Coronin 1B mRNA and protein are highly expressed in human pulmonary artery, umbilical vein, aortic and lung microvascular endothelial cells (**Figure 1** **A & B**). Under normal growth conditions, as evidenced by immunocytochemistry, coronin 1B co-localizes with F-actin in a ∼2 µM thick region at the leading edge of the cell periphery (**Figure 2**). This is presumably the fast “tread-milling” region of F-actin polymerization that has been well-characterized for cell lamellipodia. Furthermore, a significant fraction of coronin is also diffusely distributed within the cell cytoplasm, but this population of coronin does not co-localize with F-actin or cortactin. Upon serum starvation, coronin redistributes from the cell periphery and is distributed only within the cell cytosol (**Figure 3**).

### S1P Stimulates Accumulation of Coronin 1B and Cortactin to Lamellipodia

**Figure 4:**
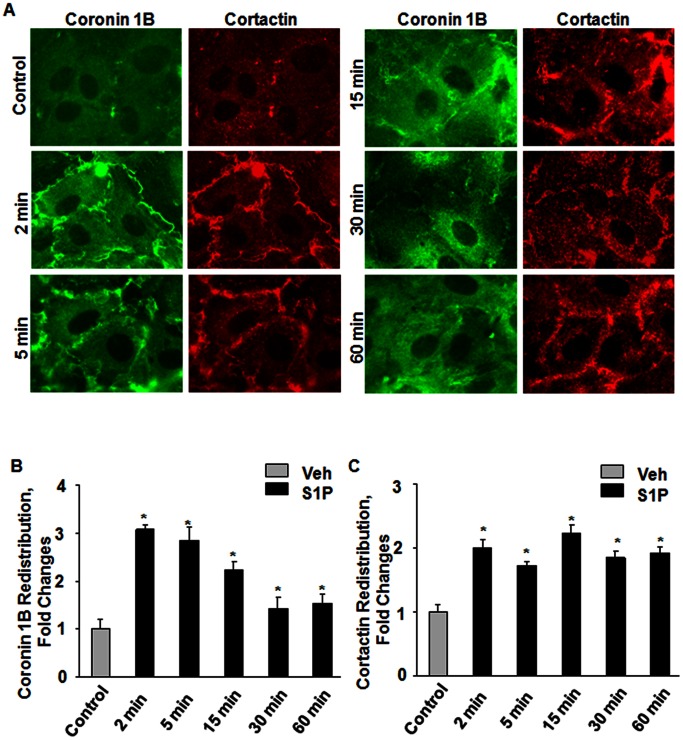
S1P stimulates redistribution of coronin 1B and cortactin to lamellipodia in human lung endothelial cells. HPAECs grown on slide chambers (∼90% confluence) were stimulated with 1 µM S1P for different time interval (2, 5, 15, 30 and 60 min) as indicated. Redistribution of Coronin 1B (**B**) and Cortactin (**C**) was visualized by immunocytochemistry and quantified by ImageJ software as described in Materials and Methods. Shown are representative immunofluorescence images from several independent experiments.

S1P is a potent angiogenic factor present in plasma at nM to µM levels [27], [28] and an activator of endothelial signal transduction [29], [30], [31]. Stimulation of HPAECs with S1P (1 µM) resulted in a rapid (2–30 min) and transient accumulation of coronin 1B in membrane ruffles (**Figure 4** **A**) at the leading edge of the lamellipodia (**Figure 4** **B**). In addition to coronin 1B, S1P also stimulated redistribution of cortactin to lamellipodia (**Figure 4** **A and C**).

### S1P Stimulated HPAEC Chemotaxis is Coronin 1B-depedent

**Figure 5:**
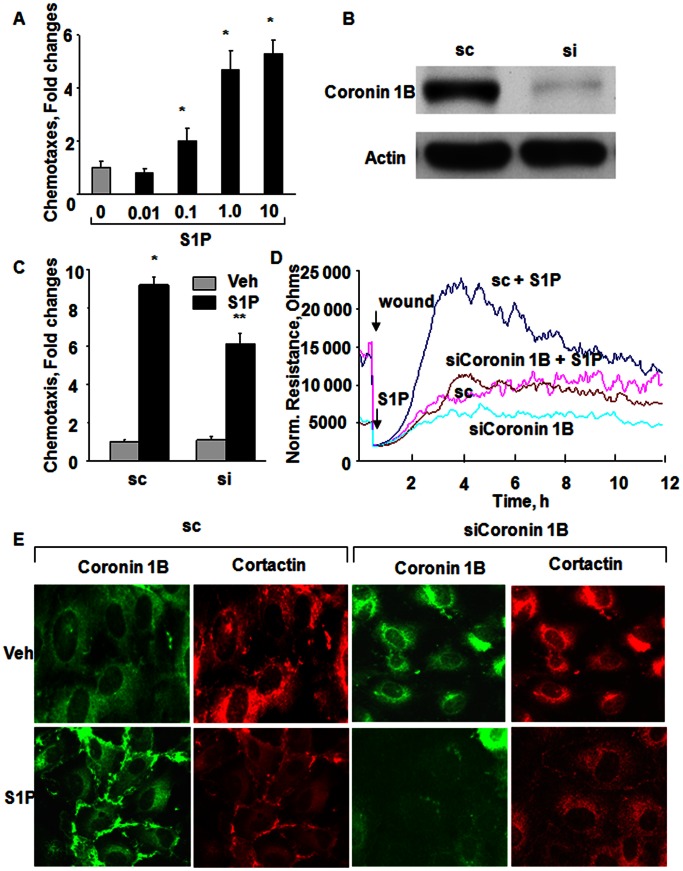
Coronin 1B siRNA attenuates S1P-induced chemotaxis, wound healing and lamellipodia localization of cortactin in HPAECs. (**A**), HPAECs grown on transwell inserts were stimulated with different S1P concentration (0.01, 0.1, 1 and 10 µM) for 15 min and chemotaxis was estimated by a Boyden chamber-based trans-well assay as described in Materials and Methods. The values are mean±SEM of three independent experiments. *, p<0.05 compared to cells without S1P. (**B**) HPAECs were transfected with scrambled (sc) or siRNA for Coronin 1B (50 ng/ml, 72 h), and cell lysates (20 µg of protein) were subjected to 10% SDS-PAGE and probed with Coronin 1B and actin antibodies as indicated. (**C**) HPAECs grown to 50% confluence in 100-mm dishes were transfected with sc (sc) or Coronin 1B siRNA (50 ng/ml) for 72 h. The cells were trypsinazied and plated on to transwell inserts and S1P-induced chemotaxis was determines as described in (**A**). The values are mean±SEM of three independent experiments in triplicate. *, p<0.05 compared cells without S1P; **, p<0.001 compared to scrambled siRNA transfected cells plus S1P. (**D**), HPAECs transfected with scrambled (sc) or Coronin 1B siRNA (50 nM, 72 h) were wounded on the gold electrodes as described under Materials and Methods. Measurement of transendothelial electrical resistance (TER) using an electrical cell substrate impedance-sensing system (ECIS) for 12 h after wounding the cells on the gold electrode and exposure to 1.0 µM S1P was carried out. Shown is a tracing from three independent experiments in triplicate. (**E**), HPAECs transfected with scrambled (sc) or Coronin 1B siRNA (50 nM, 72 h) were seeded on slide chambers for 24 h prior to stimulation with 1 µM S1P for 15 min. Cells were fixed and Coronin 1B and Cortactin redistribution to cell periphery was visualized by immunocytochemistry as described in Materials and Methods. Shown is a representative immunofluorescence image taken using an X 60 oil objective as described under Materials and Methods.

Having established that S1P stimulates redistribution of coronin1B to cell periphery, next we investigated the role of coronin1B in endothelial cell (EC) chemotaxis. Treatment of HPAECs with S1P induced cell chemotaxis in a Boyden chamber-based trans-well assay with increased cell transmigration observed at S1P concentrations of 0.1 µM and reaching a plateau at 1 µM (**Figure 5** **A**). Down-regulation of coronin 1B expression by coronin 1B small interfering RNA (siRNA) (50 nM, 72 h) knocked down >85% of coronin 1B expression (**Figure 5** **B**) and inhibited S1P-induced endothelial chemotaxis (∼36%) and cell migration (∼85%) in a wound healing assay (**Figure 5** **C and D**). This divergent result between chemotaxis and migration may be explained by inherent differences in the time frame of exposure and presentation of S1P to endothelial cells. In the chemotaxis assay, endothelial cells were allowed to migrate through a membrane filter for 6 h against a S1P gradient while in the migration assay, cells were allowed to migrate for 12–16 h in the presence of S1P that was added on to top of the cells. Further, S1P stimulated redistribution of coronin 1B and cortactin to lamellipodia compared to scrambled siRNA (Coronin 1B: = Control, 1±0.2, S1P = 3.7±0.1; Cortactin: Control, 1±0.4, S1P, 3.2±0.1); however, coronin 1B siRNA did not affect S1P-induced cortactin translocation to the lamellipodia (Control = 1±0.4; S1P = 3.2±0.1; siRNA = 1.5±0.2; siRNA+S1P = 2.0±0.1) (**Figure 5** **E**). These results suggest a role for coronin 1B in S1P mediated chemotaxis of lung endothelial cells.

### PLD2, but not PLD1, Regulates S1P-induced Coronin 1B Translocation to Cell Periphery and Chemotaxis of HPAECs

**Figure 6:**
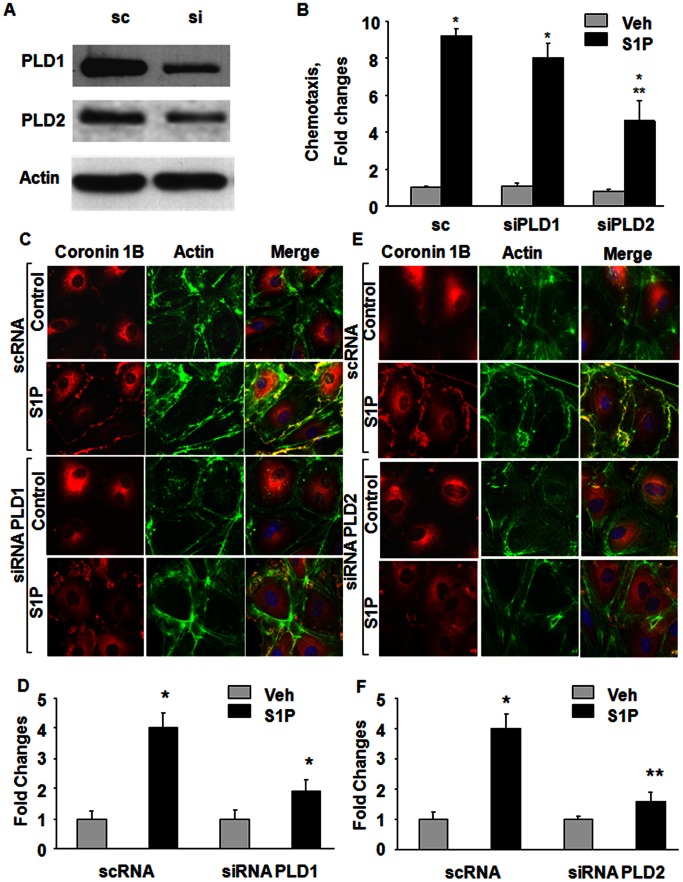
Role of PLD2 in S1P-induced chemotaxis, Coronin 1B and actin lamellipodia localization in HPAECs. HPAECs (∼50% confluence) were transfected with scrambled (sc), PLD1 or PLD2 siRNA (50 ng/ml) for 72 h. (**A**) Cell lysates (20–40 µg of protein) were subjected to 10% SDS-PAGE, Western blotted and probed with PLD1 and PLD2 antibodies as indicated; (**B**) chemotaxis of scrambled (sc) or siRNA transfected cells to S1P (1 µM) for 15 min was carried out in a Boyden chamber-based trans-well assay as described under Materials and Methods. Values are mean±SEM of three independent experiments in triplicate. *, p<0.01 compared cells without S1P; **, p<0.005 compared to scrambled siRNA transfected cells plus S1P; HPAECs transfected with sc, PLD1 (**C**) or PLD2 (**E**) siRNA in 100-mm dishes as described under (**A**) were trypsinazied and seeded onto slide chambers prior to stimulation with S1P (1 µM) for 15 min. Cells were washed, fixed, permeabilized, and probed with anti-Coronin 1B and AlexaFluor Phalloidin antibodies, and redistribution of Coronin 1B and actin due to downregulation of PLD1 (**D**) or PLD2 (**F**) was examined by immunofluorescence microscopy using a 60 X oil objective and quantified by ImageJ software as described under “Experimental Procedures”. Shown is an immunofluorescence micrograph from three independent experiments.

We have earlier demonstrated that S1P activates PLD1 and PLD2 in human bronchial epithelial cells [32], [33] and human lung ECs [15]. To determine the role of PLD1 and PLD2 in S1P-mediated coronin 1B translocation to cell periphery, and chemotaxis, HPAECs were transfected with scrambled, PLD1 or PLD2 siRNA (50 nM, 48 h) prior to S1P (1 µM) treatment. In scrambled siRNA treated cells, S1P stimulated [^32^P]PBt accumulation, an index of PLD activation [32], [33] ∼4 fold (vehicle, 1089±124 dpm; S1P, 7504±234 dpm). Downregulation of PLD1 (PBt formed: PLD1 siRNA, 965±158 dpm; PLD1 siRNA+S1P, 3168±198 dpm) or PLD2 (PBt formed: PLD2 siRNA, 690±176 dpm; S1P, 2166±122 dpm) with siRNA partially attenuated S1P-induced [^32^P]PBt formation without altering basal activity. In cells transfected with PLD1 or PLD2 siRNA, the efficacy of knocking down the protein was ∼80% compared to scrambled siRNA treated cells (**Figure 6** **A**). Downregulation of PLD2, but not PLD1, by siRNA attenuated both S1P-induced endothelial chemotaxis (∼45%) **(** **Figure 6** **B).** Knockdown of PLD2, but not PLD1, with siRNA blocked S1P-induced coronin 1B and actin translocation to lamellipodia (**Figure 6** **C–F**). These results suggest a role for PLD2, but not PLD1, in S1P-induced translocation of coronin 1B to cell periphery and chemotaxis.

### Role of Rac1 in S1P-induced Coronin 1B Distribution to Cell Periphery and Chemotaxis of HPAECs

**Figure 7:**
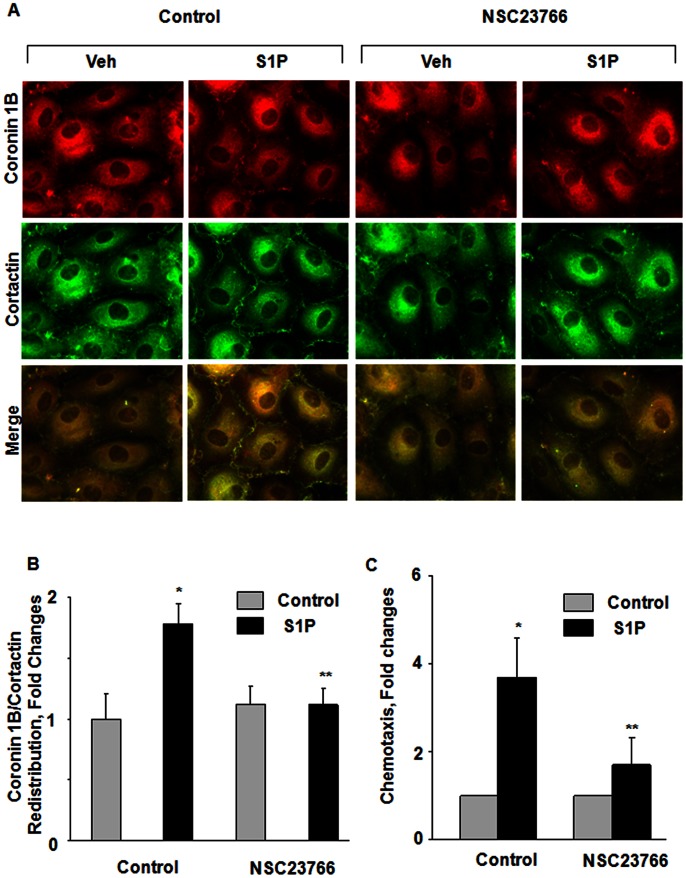
Role of Rac1 in S1P-induced chemotaxis and redistribution of coronin 1B and cortactin to lamellipodia in human lung endothelial cells. (**A**), HPAECs (∼90% confluence) grown on chamber slides were pretreated for 30 min with NSC23766 (50 µM), a Rac1 inhibitor, prior to stimulation with S1P (1 µM) for 15 min. Cells were washed, fixed, permeabilized, probed with antibodies, and redistribution of Coronin 1B and Cortactin was examined by immunofluorescence microscopy using a 60 X oil objective and quantified by ImageJ software (**B**) as described under Materials and Methods. Shown is an immunofluorescence micrograph from three independent experiments. (**C**), In parallel experiments the effect of NSC23766 on chemotaxis was determined by a Boyden chamber-based trans-well assay as described in Materials and Methods. Values are mean±SEM of three independent experiments. *, p<0.05 compared cells without S1P; **, p<0.005 compared to cells stimulated with S1P in the absence of NSC23766.

We have previously reported that PLD2 regulates S1P-induced HPAEC migration via Rac1 [15]. The role of Rac1 in S1P-induced chemotaxis and coronin 1B translocation to cell periphery is unclear; therefore, we determined whether Rac1 is involved S1P-induced chemotaxis and coronin 1B localization to lamellipodia. S1P (1 µM) stimulated the translocation of Rac1 to lamellipodia **(** **Figure 7** **A and B)**, which was blocked by NSC23766, an inhibitor of Rac1 [34], [35]. Further, S1P-induced coronin 1B translocation to cell periphery and chemotaxis was also attenuated by Rac1 inhibitor NCS23766 (**Figure 7** **A, B and C**). In addition to coronin 1B, the Rac1 inhibitor also blocked S1P-mediated translocation of cortactin to cell plasma membrane (**Figure 7** **A and B**). These results suggest a role for Rac1 in S1P-induced redistribution of coronin 1B and cortactin to cell periphery as well as chemotaxis of HPAECs.

### Role of PLD2 in S1P-induced Rac1 Activation

**Figure 8:**
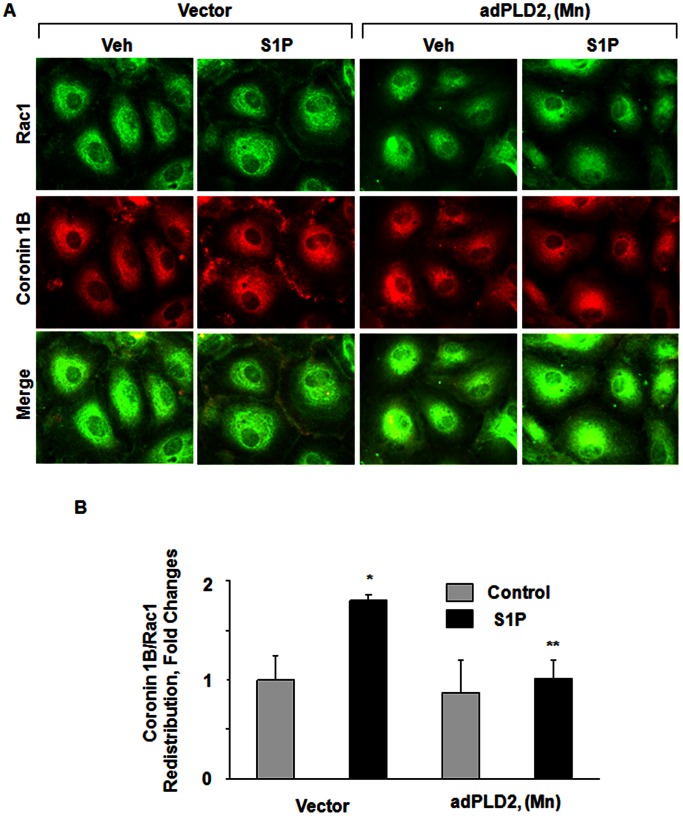
PLD2 mutant attenuates S1P-induced lamellipodial localization of Rac1 and Coronin 1B in human lung endothelial cells. HPAECs (∼50% confluence) grown on slide chambers were infected with vector-control or adenoviral mPLD2 K758R mutant (5 MOI) for 24 h, prior to stimulation with 1 µM S1P for 15 min. Cells were washed, fixed, permeabilized, probed with antibodies, and redistribution of Rac1 and Coronin 1B was examined by immunofluorescence microscopy using a 60 X oil objective and quantified by ImageJ software (**B**) as described under Materials and Methods. Shown are representative immunofluorescence micrographs from three independent experiments.

Having demonstrated a role for PLD2 and Rac1 in S1P-induced coronin 1B translocation to lamellipodia and chemotaxis, next we investigated the role of PLD2 in S1P-induced Rac1 activation. HPAECs grown on glass cover slips were infected with vector control or adenoviral mPLD2 mutant K758R (5 MOI, 24 h). Over-expression of mPLD2 mutant in HPAECs attenuated S1P-induced translocation of Rac1 to lamellipodia and decreased S1P-induced association of Rac1 with coronin 1B (**Figure 8** **A and B**). These results further establish that S1P-induced coronin 1B translocation to lamellipodia is through PLD2-Rac1 signaling cascade.

### Role of PKC Isoforms on S1P Mediated Chemotaxis of HPAECs

**Figure 9:**
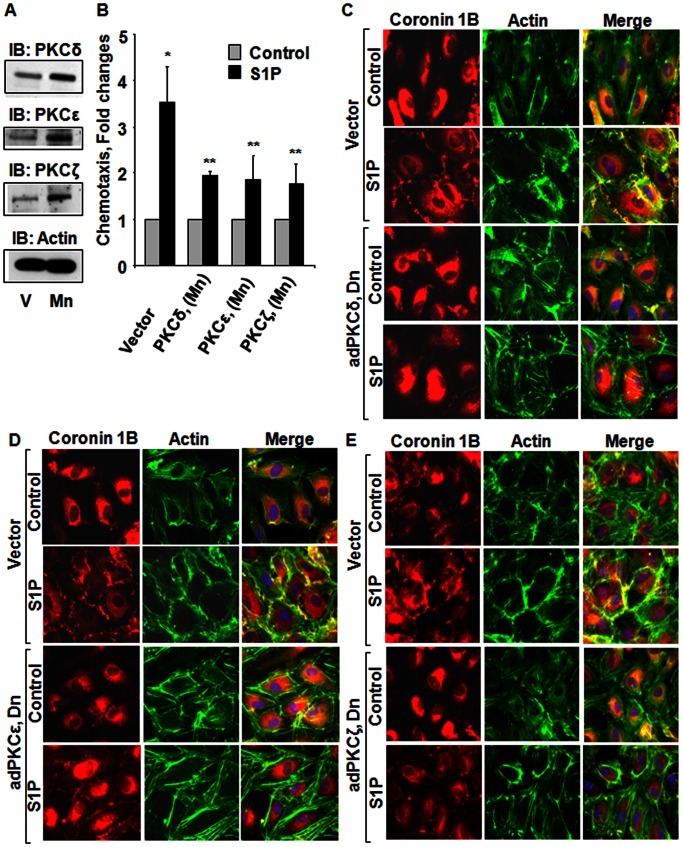
Role of PKC δ, ε, and ζ isoforms on S1P mediated chemotaxis and lamellipodial localization of Coronin 1B in human lung endothelial cells. HPAECs grown on slide chambers or 35-mm dishes (∼70% confluence) were infected with empty vector or adenoviral vectors encoding PKC dominant negative (dn) δ, ε, and ζ isoforms (5 MOI) in complete EGM-2 medium for 24 h. (**A**), Cell lysates (20 µg of protein) were subjected to 10% SDS-PAGE, Western blotting and probed with anti-PKC δ, ε, ζ and actin antibodies. In parallel experiments, the effect of dn PKC δ, ε and ζ isoforms on chemotaxis (**B**) and lamellipodial localization of coronin 1B and actin (**C, D and E**) was examined as described in Materials and Methods. Values are mean±SEM of three independent experiments. *, p<0.01 compared cells without S1P; **, p<0.005 compared to cells infected with empty vector and stimulated with S1P.

After establishing a role for PLD2 and Rac1 in S1P-mediated coronin 1B redistribution to cell periphery and chemotaxis of HPAECs, next we determined the role of PKC δ, ε, and ζ isoforms in S1P-induced coronin 1B translocation to cell periphery and chemotaxis. Challenge of HPAECs with S1P (1 µM) activated PKC δ, ε, and ζ isoforms [15]. To investigate the role of PKC δ, ε, and ζ isoforms on S1P-stimulated coronin 1B translocation and chemotaxis, HPAECs were infected with adenoviral vectors encoding for dominant negative (dn) δ, ε, and ζ (5 MOI) for 24 h, which resulted in over-expression of each of the isoform protein (∼3–5 fold) (**Figure 9** **A**). Over-expression of dn PKC δ, ε, and ζ isoforms significantly reduced S1P-induced chemotaxis (**Figure 9** **B**) and redistribution of coronin 1B and actin to cell periphery (**Figure 9** **C, D and E**) compared to vector-infected cells and co-localization of actin and coronin 1B was quantified (Coronin 1B and Actin Merged: Vector = 1±0.2; Vector+S1P = 4.0±0.1; adPKCδ (Dn) = 1±0.1; adPKCδ (Dn)+S1P = 1.7±0.1; adPKCε (Dn) = 1±0.1; adPKCε (Dn)+S1P = 1.3±0.2; adPKCζ (Dn) = 1±0.2; adPKCζ (Dn)+S1P = 1.9±0.2). These results establish that S1P-induced chemotaxis and coronin 1B translocation are dependent on PKC δ, ε, and ζ isoforms.

## Discussion

Directional migration or chemotaxis of endothelial cells plays a fundamental role in many physiological and pathological processes such as embryonic development, wound healing, tissue remodeling, angiogenesis, and tumor metastasis [36]. Chemotaxis of ECs is mediated by growth factors, chemokines, extracellular matrix-derived molecules and bioactive lipids such as platelet activating factor, and S1P [37], [38]. S1P mediated migration of human ECs requires activation of α_v_β_3_ and PKC-ε dependent activation of PLD2 and subsequently a PLD2→PKC-ζ→Rac1 signaling cascade [15]. The data presented here reveal that coronin 1B, an actin binding protein, regulates S1P-induced EC chemotaxis. Furthermore, we show that S1P-induced translocation of coronin 1B to lamellipodia and chemotaxis is regulated by PKC δ, ε, and ζ isoforms, PLD2 and Rac1 signaling cascade. In addition, it is well established that S1P mobilizes sequestered calcium by activating G protein-coupled receptors via the PLC → PIP_2_ → IP3 pathway, which induces a transient calcium release from the endoplasmic reticulum followed by activation of store-operated calcium entry resulting in Ca^2+^-influx from extracellular media [39]. Thus, S1P-induced intracellular calcium changes result in cytoskeletal remodeling, enhanced chemotaxis, motility, vascular maturation and angiogenesis in ECs [1], [29], [30].

Chemotaxis depends upon a cell’s coordinated management of its actin cytoskeleton and is thought to occur at the leading edge of plasma membrane of the cell and the cell then pulling itself toward this leading edge [12], [40], [41] in response to gradient-dependent extracellular stimuli such as S1P. There is considerable evidence that S1P mediated activation of Rho GTPase family including Rho, Rac and Cdc42 are involved in cytoskeletal reorganization and cell migration [38], [42], [43]. Our results show that S1P treatment leads to increased localization of Rac1 and coronin 1B at the leading edge and blocking Rac1 attenuated S1P-induced coronin 1B reorganization at the leading edge and chemotaxis. S1P mediates its action via S1P_1–5_ G-protein coupled receptors and S1P signaling via S1P_1_ stimulates chemotaxis of ECs [44], [45]. In contrast to ECs, S1P mediates chemotaxis of fibroblasts through S1P_1,3_ or S1P_2._ In human lung fibroblasts, S1P-mediated chemotaxis was through S1P_2_ [46]; however, S1P dependent chemotaxis in human primary dermal fibroblast was dependent on S1P_1,3_ [47]_._ In contrast to stimulation of chemotaxis/migration of ECs [15], [48], [49] , keratinocytes [50], glioma cells [51] and fibroblast [46], [47], S1P inhibited migration of breast cancer cells [38] and melanoma and fibrosarcoma cells [52]. This bimodal regulation of chemotaxis/migration by S1P may be due to expression of stimulatory or inhibitory S1P receptors on different cell types, concentrations of S1P used, and coupling to varying down-stream signaling molecules.

S1P stimulates PLD isoforms 1 & 2 in human lung ECs [15], [26] and both the isoforms catalyze the hydrolysis of phosphatidylcholine and other phospholipids to PA. PA acts intracellularly as a second messenger [53] and PA generated via PLD signal transduction has been shown to be involved in membrane trafficking [54], [55], actin cytoskeleton remodeling [56], NADPH oxidase activation [39], [57], [58], cytokine secretion [33] and endothelial barrier function [59], [60]. Further, several studies suggest a role for PLD1 and PLD2 in cell motility. Over-expression of catalytically inactive PLD2 inhibited migration of ECs [15], fibroblasts [61] and cancer cells [16], [62] suggesting a role for PLD2/PA signaling in regulation of cell motility. Consistent with these studies, S1P-induced migration of lung ECs was attenuated by over-expression of PLD2 (K758R), but not PLD1 (K898R), mutant in HPAECs [63]. Further, S1P-induced cell motility was dependent on intracellular S1P generation as blocking SphK1 attenuated the cell migration mediated by exogenous S1P (64).

At present, the potential explanation for the differential participation of PLD1 and PLD2 is unclear but could be due to differences in the sub-cellular localization of PLD1 and PLD2 in mammalian cells. In mammalian cells, PLD1 is localized in the cytosol, Golgi membranes, nucleus and plasma membrane while PLD2 is primarily localized in the plasma membrane [64], [65], [66]. It is unclear how PA generated by PLD2 activation regulates coronin 1B [67]. Previously, we have shown that PLD regulates Rac1 via PKC-ζ and migration of HPAECs to S1P [15]; however, mechanism(s) of PA-dependent activation of PKC-ζ is yet to be fully defined. PKC-ζ can be activated by acidic lipids including PA [68] but it is unclear if PKC-ζ has any domain structure for PA binding. Several studies have demonstrated an important role for RhoA family of GTPases, Rho, Rac, and Cdc42 in regulating cell migration in response to agonists [67], [69], [70]. It is well recognized that actin polymerization leading to the formation of stress fibers is RhoA-dependent [71], [72], a process that is partly regulated by the PLD/PA signaling axis [56], [59]. Interestingly, PLD2 has guanine nucleotide-exchange factor (GEF) activity for Rho and regulates actin stress fibers in a manner independent of its lipase activity [73], [74]. Additionally, PA activates phosphatidylinositol-4-phosphate 5 kinase (PI4P5K) [75], [76], [77] to generate phosphatidylinositol-4,5-bisphosphate (PIP_2_), an activator of actin cytoskeleton and of interactions between actin and actin-binding proteins such as vinculin and filamin [78], [79]. However, the role of PLD2-generated PA in activation of Coronin 1B via PI4P5K is not known.

Current evidence supports that Coronin 1B disassembles Arp2/3 containing actin filament branches by inducing Arp2/3 dissociation and alters the branch angle [80]. Phosphorylation of Coronin 1B at ser-2 by PKC regulates its interaction with Arp2/3 complex and reduces phorbol ester-induced motility of fibroblast [81] . However, in vascular smooth muscle cells, phosphorylation of coronin 1B at ser-2 was essential for PDGF-induced migration [82]. Although PDGF-induced phosphorylation of Coronin 1B reduced its interaction with Arp2/3 complex, an important step in inducing cell migration, the differential effect of Coronin 1B phosphorylation on cell motility mediated by phorbol ester in fibroblast and PDGF in vascular smooth muscle cells remains controversial. In lung ECs, S1P stimulated tyrosine phosphorylation of Coronin 1B while phosphorylation at ser-2 was comparatively less to tyrosine phosphorylation (V. Natarajan, unpublished results); however the role of tyrosine phosphorylation of Coronin 1B in chemotaxis remains to be established.

**Figure 10:**
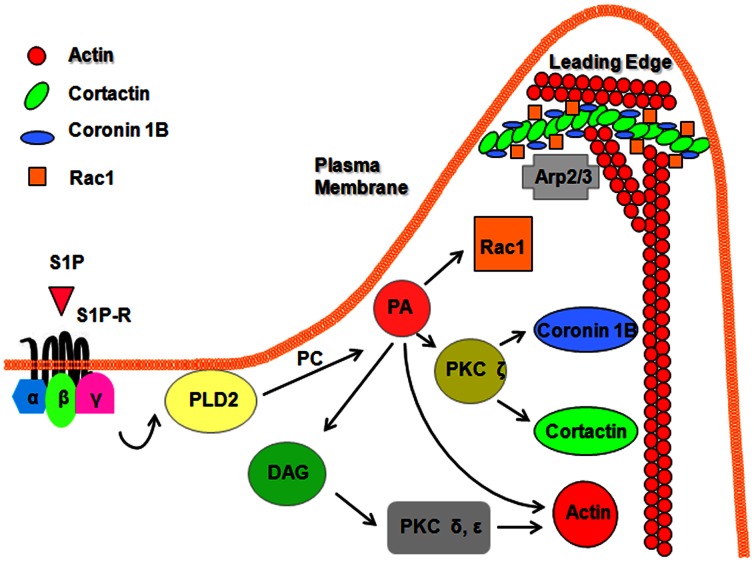
Proposed signaling mechanisms involved in S1P-induced lamellipodial localization of Coronin 1B, Cortactin and chemotaxis of human lung endothelial cells. S1P binding to G-protein coupled S1P1-5 receptors activates PLD2 via PKC δ and ε and activation of PLD2 results in hydrolysis of membrane associated phosphatidylcholine (PC) to phosphatidic acid (PA) and phospho-choline. PA can be converted to DAG by PA-phosphatases or can activate PKC ζ via of phosphatidylinositol-4-phosphate-5kinase activation. Activation of PKC ζ results in redistribution of Coronin 1B and Cortactin to cell periphery and localization in lamellipodia of endothelial cells. PA can directly bind to and activate Rac1 and formation of actin stress fibers. Additionally, PLD2 has guanine nucleotide-exchange factor (GEF) activity for Rho and can regulate actin stress fibers in a manner independent of its lipase activity. S1P-induced activation of PKC δ/ε → PLD2/PA → PKC ζ → Rac1 signaling cascade facilitates recruitment of Coronin 1B, Cortactin and Actin to lamellipodia and chemotaxis of endothelial cells.

In conclusion, the present studies demonstrate that translocation of Coronin 1B to cell periphery participates in S1P-induced chemotaxis of HPAECs. Further, our results suggest that S1P-induced phosphorylation of Coronin 1B and redistribution to cell periphery is regulated by PLD2, Rac1 and PKC isoforms ε and ζ. Taken together, these observations provide new insights into role of Coronin 1B in S1P-induced regulation of chemotaxis in lung ECs (**Fig. 10**).

## Materials and Methods

### Materials

Sphingosine-1-phosphate (S1P) was obtained from Avanti Polar Lipids (Alabaster, AL, USA). Scrambled siRNA and target siRNA for PLD1, PLD2 and Coronin1B, and antibodies for cortactin, PKC δ, ε, and ζ were obtained from Santa Cruz Biotechnology Inc. (Santa Cruz, CA, USA). Anti-coronin1B antibody was kind gift from Dr. James Bear (UNC, Chapel Hill, NC), and anti-Rac1 antibody was from BD Biosciences Pharmingen (San Jose, CA, USA). Internal and N-terminal antibodies for PLD1 and PLD2 were purchased from BioSource International Inc. (Camarillo, CA, USA), and anti-PLD2 antibody was kindly provided by Dr. Sylvain Bourgoin (Quebec, PQ, Canada). NSC23766 was from Calbiochem (San Diego, CA, USA). Transwell inserts were from Becton Dickinson Lab ware (Franklin Lakes, NJ, USA), and slide chambers were from Millipore (Bedford, MA, USA). Lysis buffer was purchased from Cell Signaling Technology Inc. (Danvers, MA, USA). Precast Tris-Glycine PAAG (Invitrogen-Molecular Probes, Eugene, OR, USA). Protease inhibitor cocktail tablets (EDTA-free Complete) were from Roche Diagnostics (Indianapolis, IN, USA). Aprotinin and phosphatase inhibitor cocktail 1, actin antibody were from Sigma-Aldrich (St. Louis, MO, USA).

### Cell Culture

HPAECs were purchased from Lonza (San Diego, CA, USA), cultured in complete endothelial growth medium (EGM)-2 medium [15]. Cells (passage number 4–6) plated in 35-mm, 100-mm dishes or slide chambers were used for all the experiments.

### Endothelial Cell Chemotaxis

HPAECs were cultured to ∼90% confluence, starved in EBM-2 medium containing 0.1% FBS for 1–3 h. Starved cells were suspended in 0.25% Trypsin-EDTA, neutralized with trypsin neutralizing solution, pelleted by centrifugation (500×g, 5 min), resuspended in EBM-2 media (0.1% serum), and counted using hemocytometer. 200 µl of cell suspension, containing 2×10^3^ cells was added to the top well of a 24-mm diameter, 8.0 µM pore size Transwell insert and 600 µl of starvation media was added to the bottom chamber. Cells were allowed to attach for 1 h on the insert, S1P was added to the bottom chamber to a final concentration of 1 µM, and cells were allowed to migrate for 6 h. Cells that did not migrate were removed from the top portion of the insert using a cotton swab and migrated cells at the bottom side of the insert were fixed submerging the insert in 3.7% paraformaldehyde. The transwell membrane was removed and sealed using Vectashield containing DAPI. Cells were visualized under UV fluorescence using a Hamamatsu digital camera connected to the Nikon Eclipse TE2000-S microscope with ×10 objective and MetaVue software (Universal Imaging Corp., PA, USA). The effect of S1P and other agents on cell chemotaxis was quantified by manual counting of DAPI-stained nuclei of cells that have migrated across the Transwell membrane.

### Electrical Cell Substrate Impedance Sensing (ECIS) Assay

HPAECs were cultured in 8-well ECIS electrode arrays (8W1E, Applied Biophysics, NY, USA) [83] to ∼95% confluence and starved in the EBM-2 medium with 0.1% BSA for 1–3 h. An elevated field (3 V at 40,000 Hz for 10 sec) was applied to wound the cells on the electrode and S1P was immediately added and endothelial wound healing was monitored for 12 h by measuring the transendothelial electrical resistance.

### Infection of HPAECs with Adenoviral Vectors

cDNA for wild type and catalytically inactive mutants of PLD1, PLD2, and dominant negative (dn) PKC δ, ε, and ζ were sub-cloned into the pShuttle-CMV vector [15], [33]. The recombinant plasmid was linearized and transfected into HEK293 cells to generate replication-defective adenovirus. Generation of purified virus [10^10^plaque-forming units (p.f.u.)/ml] was carried out by the University of Iowa Gene Transfer Vector Core. Purified adenovirus (1–10 M.O.I) in complete EGM-2 medium was added to HPAECs grown to ∼80% confluence in 6-well plates, slide chambers, 60- or 100-mm dishes. After 24 h, the virus-containing medium was replaced with complete EGM-2 medium. Vector control or infected cells were subjected to scratch and wound healing ECIS assays and immunoprecipitates or cell lysates from parallel experiments were analyzed by Western blotting.

### Western Blot Analysis

HPAECs were cultured in 6-well plates or 60-mm dishes to ∼95% confluence and starved for 3 h in EBM-2 medium containing 0.1% FBS. Cells were stimulated with S1P (100–1000 nM) for 5–60 min, washed with PBS and lysed with 100–300 µl lysis buffer containing 20 mM Tris-HCl (pH 7.5), 150 mM NaCl, 1 mM Na_2_EDTA, 1 mM EGTA, 1% Triton X-100, 2.5 mM sodium pyrophosphate, 1 mM β-glycerophosphate, 1 mM Na_3_VO_4_, 1 µg/ml leupeptin, 1 µg/ml aprotinin and protease inhibitors, EDTA-free complete tablets (Roche Applied Science, Indianapolis, IN). Cell lysates were cleared by centrifugation at 10,000×g for 10 min, and boiled with the Laemmli sample buffer for 5 min. Cell lysates (20–30 µg protein) were separated on 10% or 4–20% SDS-PAGE, transferred to PVDF membranes, blocked in TBST containing 5% BSA prior to incubation with primary antibody (1∶1000 dilution) overnight. After blocking, washing and incubation with appropriate secondary antibody (1∶2000 dilution), blots were developed using an ECL chemiluminescence kit. Western blots were scanned by densitometry and integrated density of pixels in identified areas was quantified using Image Quant version 5.2 software (Molecular Dynamics).

### Immunofluorescence and Confocal Microscopy

HPAECs grown on chamber slides were starved for 3 h in EBM-2 containing 0.1% FBS prior to treatment with S1P (100–1000 nM) for 5–60 min. Cells were fixed in 3.7% para-formaldehyde in PBS for 10 min, washed three times with PBS, permeabilized for 4 min in 3.7% paraformaldehyde containing 0.25% Triton X-100, blocked with 2% BSA in TBST, incubated for 1 h with appropriate primary antibody (1∶200 dilution), washed with TBST, and stained for 1 h with secondary antibody (1∶200 dilution) in TBST containing 2% BSA. Cells were examined using a Nikon Eclipse TE2000-S immunofluorescence microscope and a Hamamatsu digital camera with ×60 oil immersion objective and Meta Vue software (Universal Imaging Corp., PA, USA). Coronin 1B and actin redistribution to lamellipodia was also investigated using Zeiss 510 Meta laser scanning microscope. Quantification of lamellipodia was performed as described earlier [39]. Briefly, for each image, background signal was subtracted by drawing a region of interest around the cell periphery of individual cells. All areas outside the cell were cleared to best visualize the leading edges including cell periphery and the fluorescence intensity within the entire cell was quantified by MBF ImageJ bundle (Tony Collins, McMaster University, http://www.macbiophotonics.ca/imageJ/and Wayne Rasband, NIH, http://rsb.info.nih.gov/ij/).

### Transfection Procedures

HPAECs grown to ∼50% confluence in 6-well plates or chamber slides were transfected with Gene Silencer® (Gene Therapy System, Inc. San Diego, CA, USA) transfecting agent containing scrambled siRNA (50–100 nM) or siRNA for target proteins (50–100 nM) in serum-free EBM-2 medium according to manufacturer’s recommendation. To optimize conditions for efficient transfection, HPAECs were transfected with Fl-Luciferase GL2 Duplex siRNA (Target Sequence: 5′-CGTACGCGGAATACTTCGA-3′, Dharmacon, CO, USA) as a positive control. After 3 h transfection, 1 ml of fresh complete EGM-2 medium containing 10% FBS was added, cells were cultured for additional 72 h, and analyzed for mRNA level by real time PCR or protein expression by Western blotting.

### PLD Activation in Intact Lung Endothelial Cells

HPAECs were labeled with [^32^P] orthophosphate (5 µCi/ml) in phosphate-free medium containing 2% FBS for 18–24 h. Cells were washed in minimal essential medium without serum and challenged either with vehicle or S1P (1 µM) for 30 min in the presence of 0.05% 1-butanol or tertiary butanol. The incubations were terminated by addition of 1ml of methanol-concentrated HCl (100∶1/vol/vol), followed by extraction of lipids [26]. [^32^P]PBt formed as a result of PLD activation and transphosphatidylation reaction, an index of *in vivo* PLD stimulation [84], was separated by TLC on 1% potassium oxalate-impregnated silica gel H plates using the upper phase of ethyl acetate-2,2,4-trimethyl pentane-glacial acetic acid-water (65∶10∶15∶50 vol/vol/vol/vol) as the developing solvent system [26]. Unlabeled PBt was added as a carrier during the lipid separation by TLC and was visualized under iodine vapors. Radioactivity associated with PBt was quantified by liquid scintillation counting, and data are expressed as dpm normalized to 10^6^ counts in total lipid extract.

### Statistical Analysis

Analysis of variance and Student-Newman-Keul’s test were used to compare means of two or more different treatment groups. The level of significance was set to p<0.05 unless otherwise stated. Results are expressed as mean ± S.E. M.
